# Prevalence of HPV infection among Greek women attending a gynecological outpatient clinic

**DOI:** 10.1186/1471-2334-10-27

**Published:** 2010-02-15

**Authors:** Petroula Stamataki, Athanasia Papazafiropoulou, Ioannis Elefsiniotis, Margarita Giannakopoulou, Hero Brokalaki, Eleni Apostolopoulou, Pavlos Sarafis, George Saroglou

**Affiliations:** 1 Surgical Department, Naval Hospital of Athens, 70 Dinokratous, 115 21 Athens, Greece; 2 3rdDepartment of Internal Medicine and Center of Diabetes, General Hospital of Nikaia "Ag. Panteleimon", 3 D.Mantouvalou, 184 54 Nikaia, Greece; 3 Department of Internal Medicine, "Elena Venizelou" Hospital, Faculty of Nursing, University of Athens, 2 E.Venizelou, 115 21 Athens, Greece

## Abstract

**Background:**

Human papillomavirus (HPV) infection is a causative factor for cervical cancer. Early detection of high risk HPV types might help to identify women at high risk of cervical cancer. The aim of the present study was to examine the HPV prevalence and distribution in cervical smears in a sample of Greek women attending a gynecological outpatient clinic and to explore the determinants of the infection.

**Methods:**

A total of 225 women were studied. All women underwent a regular gynecological control. 35 HPV types were studied; 6, 11, 16, 18, 26, 31, 33, 35, 39, 40, 42, 43, 44, 45, 51, 52, 53, 54, 56, 58, 59, 61, 62, 66, 68, 70, 71, 72, 73, 81, 82, 83, 84, 85 and 89. Also, basic demographic information, sociodemographic characteristics and sexual behavior were recorded.

**Results:**

HPV was detected in 22.7% of the study population. The percentage of the newly diagnosed women with HPV infection was 17.3%. HPV-16 was the most common type detected (5.3%) followed by HPV-53 (4.9%). 66.2% of the study participants had a Pap test during the last year without any abnormalities. HPV infection was related positively with alcohol consumption (OR: 2.19, 95% CI: 1.04-4.63, P = 0.04) and number of sexual partners (OR: 2.16, 95% CI: 1.44-3.25, P < 0.001), and negatively with age (OR: 0.93, 95% CI: 0.87-0.99, P = 0.03), and monthly income (OR: 0.63, 95% CI: 0.44-0.89, P = 0.01).

**Conclusion:**

The prevalence of HPV in women attending an outpatient clinic is high. Number of sexual partners and alcohol consumption were the most significant risk factors for HPV infection, followed by young age and lower income.

## Background

Cervical cancer is the second most common cancer in women worldwide [1]. Several studies have strongly implicated human papillomavirus (HPV) infection as a causative factor in the development of cervical cancer [2,3]. Based on their association with cervical cancer, HPV can be grouped to high-risk (such as HPV-16, -18, -31, and -45) and low-risk HPV types (such as 6, 11, 42, 43, and 44) [4]. High-risk HPV types are present in over 99% of cervical cancers and in the vast majority of cases of high grade cervical intraepithelial neoplasia [5,6]. Worldwide, approximately, 70% of cervical cancers are due to HPV types 16 and 18 [3].

Women with normal cervical cytology who are infected with high risk HPV type have an approximately 100-fold increased risk of developing cervical cancer compared to uninfected women [7]. Therefore, it has been suggested that high risk HPV detection might be used as a tool to identify women at high risk of cervical cancer, in addition to Pap smears [7,8]. Furthermore, the development of HPV vaccines and implementation of vaccination programs might help to reduce the burden of disease [9]. Especially, vaccination against HPV types 16 and 18 potentially prevents more than two thirds of cervical cancer cases worldwide. However, the impact of an HPV vaccination in different geographical regions will be related to the prevalence of HPV types 16 and 18 in the different populations [10].

Since, the prevalence of the high risk HPV types varies among different populations we conducted the present study in order to examine the HPV prevalence and distribution in cervical smears in a sample of Greek women attending a gynecological outpatient clinic and to explore the determinants of the infection.

## Methods

### Population

The study population consisted of a consecutive sample of 225 women attending the gynecological outpatient clinic of the Maternal and Perinatal Hospital "Elena Venizelou" between October 2007 and May 2008, for regular gynecological control. "Elena Venizelou" Hospital is a tertiary maternal hospital responsible for the greater region of Athens, the capital of Greece. Pregnant women or women with a recent delivery were not enrolled into the study. The refusal rate was low; only 2 women denied taking part into the study.

Basic demographic information, sociodemographic characteristics, medical history, smoking status, alcohol consumption and sexual and reproductive behaviour was obtained at the time of the gynecological visit by a study nurse. Additional questions were asked on age at first sex, number of lifetime partners, past history of sexually transmitted infections, and use of a condom.

Participants were considered as newly diagnosed cases for HPV infection if they had a negative history for HPV infection according to their medical records. In addition, newly diagnosed cases included women who had never been screened for HPV infection. Subjects were considered as non-smokers if they have never smoked or if they had given up smoking for at least three consecutive years. Heavy drinking was defined as consuming an average of more than 1 drink per day. Participants were stratified according to their monthly income and education level as it is stated at Additional file 1, Table S1. Females whose monthly incomes did not exceed 1,000 Euros were considered as low income. High education level was defined as attending a college or a university.

All subjects underwent their scheduled examination, which included the placement of a speculum in the vagina, visualization of the cervix, and collection of cervical cells using a wooden Ayre spatula and endocervical brush. After the preparation of a standard cervical smear, the remaining cells were placed in tubes with 0.9% saline and stored at -20°C prepared for HPV molecular analysis. Pap smears were classified according to the Bethesda system by local cytopathologists.

The Hospital's ethical committee provided approval for this study. All participants gave their written consent before enrollment to the study. The study was in accordance with the Helsinki declaration.

### HPV DNA Detection Techniques

#### Nucleic acid isolation

The cervical cells removed from the swabs and centrifuged in order to be in a pellet form. The supernatant discarded and the remaining cells were dissolved in 100 μl 1× Phosphate Buffer Saline (PBS). The dissolved cells transferred to 200 μl Tissue Lysis Buffer and 20 μl Proteinase K according the instruction manual of CLINICAL ARRAY 'S (HPV) (Biogenomica Spain). The extracted DNA eluted in 100 μl Elution buffer (Biogenomica Spain) and stored in -20°C. The sensitivity and specificity of the CLINICAL ARRAY' S HPV kit is 92.3% and 89%, respectively [11].

#### PCR Amplification

Five microliters (5 μl) of eluted DNA were used in the PCR amplification of pre alliquoted ready to use vials-kept at -20°C in alliquots (Biogenomica Spain). The PCR reaction includes two internal controls in order to avoid the false negative results from the extraction procedure and from the PCR inhibitors. The first amplifies a 892 bp fragment of the human CFTR gene and indicates the DNA existence and the second is a 1202 bp fragment from a modified plasmid and indicates if there is inhibitors or not.

#### Hybridization

The PCR product denaturated in 95°C for 15 minutes in iQ Thermocycler and then thawed in 4°C. Five microliters (5 μl) of the PCR product proceeded in hybridization on the specific Array Tubes according the instruction manual (Biogenomica Spain) on the ATS Workstation (CLONDIAG Chip Technologies GMBH). The hybridization results were interpreted by the Specific CLINICAL ARRAYS Human Papilloma virus software program which it has been preinstalled and ready to use on the ATS Workstation (CLONDIAG Chip Technologies GMBH).

The following 35 HPV types were studied; 6, 11, 16, 18, 26, 31, 33, 35, 39, 40, 42, 43, 44, 45, 51, 52, 53, 54, 56, 58, 59, 61, 62, 66, 68, 70, 71, 72, 73, 81, 82, 83, 84, 85 and 89. The oncogenic risk of the HPV types detectable with CE marked and IVD marked kit of Genomica are according to the Munoz et al [12].

A recent meta-analysis examined the association between detection of HPV DNA and high-grade cervical intraepithelial neoplasia and invasive cervical cancer, showing a strong association [13].

### Statistical Analysis

Statistical analysis was preformed using programs available in the SPSS statistical package (SPSS 15.0, Chicago, USA). All variables were tested for normal distribution of the data. Data are shown as mean ± SD, unless it is stated otherwise. A two sample *t*-test was used to assess differences in continuous variables, while a chi-square test was used for categorical variables. Univariate binary logistic analysis was performed to look for the relationship between HPV infection and the variables of interest in the sample population. Then, multivariate analysis was performed (backward stepwise method) to look for independent associations between HPV infection and the variables of interest. All independent variables in the multivariate analysis were tested for multicolinearity. P < 0.05 (two-tailed) was considered statistically significant.

## Results

### Characteristics of the study population

The characteristics of the study population are summarized in Additional file 1, Table S1. The study population consisted of 225 women aged 16 to 45 years (mean age ± standard deviation: 30.7 ± 6.8 years). Most of the participating women had high (68.4%) educational level, were unmarried (52.4%), had their first sexual intercourse before the age of 20 (67.1%), reported a single regular sexual partner during the last year (76.4%). Of the married women 19.5% had at least two full term pregnancies. 55.1% of the participants reported more than 3 sexual partners during their lifetime, and 29.3% had more than 5 sexual intercourses monthly. 45.3% of the study participants had high monthly income.

Condom was the most common contraceptive method ever used (58.6%). It is noteworthy that almost the one third of the study participants did not use any contraceptive method (34.2%). Of the study population, 10.6% had a history of a previous HPV infection and 48% a history of sexually transmitted disease. 8.0% had never done a Pap test, while 66.2% had a Pap test during the last year without any abnormalities. Only one third of the women were smokers (39.1%), and 48.8% reported regular alcohol consumption.

### Characteristics of women positive for HPV infection

The age of HPV-positive women (n = 51) was (mean age ± standard deviation) 28.5 ± 6.7 years. Condom was the most common contraceptive method ever used (60.8%), while 29.4% did not use any contraceptive method. Of the HPV-positive women, 23.5% had a history of a previous HPV infection and 54.9% a history of sexually transmitted disease. 13.7% had never done a Pap test, while 58.8% had a Pap test during the last year. 55.0% of the HPV-positive women were smokers and 62.8% reported regular alcohol consumption (Additional file 1, Table S1).

### HPV prevalence and subtypes

HPV was detected in 51 of the 225 women examined (22.7%) (6 positive for HPV-6, 12 for HPV-16, 2 for HPV-18, 1 for HPV-31, 2 for HPV-33, 2 for HPV-39, 3 for HPV-51, 8 for HPV-53, 2 for HPV-58, 2 for HPV-61, 1 for HPV-62, 3 for HPV-66, 3 for HPV-81, 2 for HPV-83 and 2 for HPV-84). The percentage of the newly diagnosed women with HPV infection was 17.3% (39/225). In addition, it must be mentioned that 12 women with a previous history for HPV infection were found in the HPV negative subsample. These cases were included in the HPV-negative group for the further analysis.

16.4% (37/225) of the study population was infected with a single type and 6.2% (14/225) was infected with at least two HPV types. In the entire study population, HPV-16 was the most common type detected (5.3%) either as a single type or in combination with other types, followed by HPV-53 (4.9%). It is noteworthy that the prevalence of oncogenic HPV-18 was low (0.9%). Oncogenic (HPV 16, 18, 31, 33, 39, 51, 53, 58) and nononcogenic (HPV 6, 61, 62, 66, 81, 83, 84) types were detected in 14.2% and 8.2% of the HPV-positive women, respectively.

**Figure 1:**
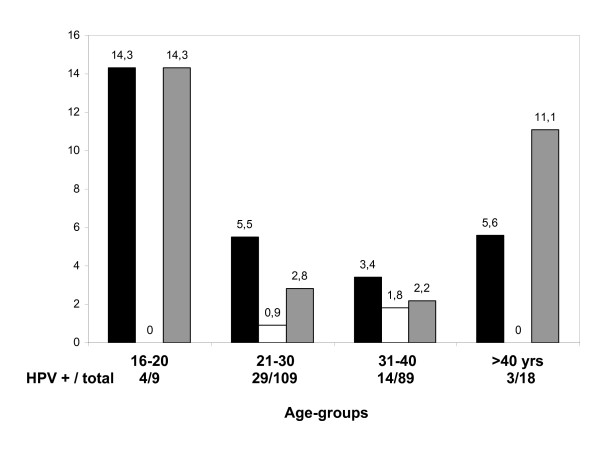
**The prevalence of HPV-16, HPV-18 and HPV-53 according to the age groups (the values are shown as %)**. "black square", HPV-16; "white square", HPV-18; "grey square", HPV-53.

The prevalence of HPV infection among females aged 21 to 30 years was 26.6% whereas the corresponding prevalence among females aged 16 to 20 years was 57.1%. In detail, the prevalence of HPV-16 and HPV-53 among females aged 21 to 30 years old were 5.5%, 2.8%, respectively, whereas the corresponding prevalences among females aged 16 to 20 years were 14.3% and 14.3%, respectively (Figure 1).

### Risk factors for HPV infection

The univariate logistic analysis showed significant associations between HPV infection and age [odds ratio (OR): 0.94, 95% Confidence Intervals (95% CI): 0.89-0.98], monthly income (OR: 0.65, 95% CI: 0.48-0.89), marital status (OR: 0.37, 95% CI: 0.18-0.73), number of full term pregnancies (OR: 0.58, 95% CI: 0.36-0.94), number of sexual partners (OR: 2.17, 95% CI: 1.50-3.15), smoking status (OR: 1.42, 95% CI: 1.08-1.85), and alcohol consumption (OR: 2.27, 95% CI: 1.18-4.35). No any significant relationships were found between HPV infection and educational level, nationality, methods of contraception, age at first sexual intercourse, and history of previous HPV infection or other sexual transmitted diseases (Additional file 2, Table S2).

Multivariate analysis demonstrated, after controlling for smoking, marital status, and number of full term pregnancies, that HPV infection was related positively with alcohol consumption (OR: 2.19, 95% CI: 1.04-4.63) and number of sexual partners (OR: 2.16, 95% CI: 1.44-3.25), and negatively with age (OR: 0.93, 95% CI: 0.87-0.99) and monthly income (OR: 0.63, 95% CI: 0.44-0.89) (Additional file 2, Table S2).

## Discussion

Prevalence of HPV DNA in a sample of Greek females aged 16 to 45 years was 22.7%, with the highest prevalence (57.1%) among women aged 16 to 20 years.

The percentage of the newly diagnosed women with HPV infection was 17.3%. Oncogenic HPV types were detected in 14.2% of the HPV-positive women. The overall prevalence of high risk HPV-16, HPV-53 and HPV-18 were 5.3%, 4.9% and 0.9% respectively. Similar prevalence of HPV-positive women has been reported in a large sample of US females aged 14 to 59 years (26.8%) [14]. Other studies in Europe have yielded similar findings in healthy women [15] as well as women with history of cervical cancer [16]. A recent study in our country demonstrated that HPV DNA was positive in 23.6% of cytologically normal women [17]. Similar rates have been confirmed by a previous study in malignant biopsies in Greece [18] and even higher rates in another study in asymptomatic women [19]. However, a study in healthy women by Agorastos et al. reported one of the lowest HPV prevalence ever observed internationally (2.5%) [20]. Also, low prevalence of HPV-positive women has been reported in studies from Spain [21] and Vietnam [22].

HPV-16 was the most prevalent oncogenic type either as a single type or in combination with other types, while the prevalence of HPV-18 was low. A lot of studies in different populations have shown that HPV-16 was the most common among the others HPV types [22-25]. Even higher prevalence of HPV-16 has been described in different geographic regions ranging from 43.9% in Asia to 72.4% in Africa in women with invasive cervical cancer [12]. Furthermore, it is known that HPV-16 represents the most commonly identified HPV type in low- and high-grade cervical lesions as well as cervical cancer worldwide [26]. However, a low prevalence of HPV-16, HPV-53 and HPV-18 has been reported in the USA [14]. The prevalence of HPV-18 (0.8%) was similar to that of the present study [14]. On the contrary, studies from our country using histological samples have reported higher prevalence of HPV-18 [18,27,28]. A study by Kroupis et al., in a sample of Greek women with a history of cervical lesions reported a higher prevalence of HPV-53 [17].

Of the total study population, 16.4% was infected with a single type and 6.2% was infected with at least two HPV types. The observed prevalence of multiple infections is low in comparison with the results from studies from different countries [29-35]. The presence of multiple infections was 0% in Brazil [29], 5.3% in Morocco [30], 9.8% in Thailand [31], 14.3% in Philippines [32], 16.7% in Paraguay [33], 28% in The Netherlands [34], and 39% in Costa Rica [35]. In our country a recent study showed that the prevalence of multiple infections was 21.2% [17]. These differences in prevalence of multiple infections could be due to differences in the technique used or real differences in the prevalence of the HPV types in the populations studied.

The results of the multivariate analysis demonstrated that HPV infection was related positively with alcohol consumption and number of sexual partners, and negatively with age and monthly income. A lot of previous studies have showed that the main risk factor for HPV infection was the number of sexual partners [36-38]. Also, alcohol consumption was an important risk factor. A simply explanation is that alcohol consumption might lead to unsafe sexual behavior and to less adherences to contraceptive methods. However, the literature data regarding the potential relationship between alcohol consumption and HPV infection are conflicting. A previous study showed that there is a positive relationship between alcohol and HPV infection [39], while a recent one showed no relationship [40]. On the contrary, HPV infection declined with increasing age. Importantly, other studies also reported the increaased HPV infection with aging, suggesting the biological effect, such as HPV immunity acquired over time [35,38,41]. The same pattern was observed in a previous study from our country [20]. Finally, low monthly income was a significant determinant of HPV infection. Two studies in the USA have showed that high-risk HPV infection is common in poor women [42,43].

Our study has some limitations. As it is mentioned above, 12 women with a previous HPV infection were found in the HPV-negative subgroup. This is partially explained by the results of two studies showing that a single negative HPV test result could lead to missed HPV infection [44] and that HPV test has low specificity [45]. In addition, the number of the participants as well as the age range in our study sample was relatively limited; only women aged 16-45 years were included. Therefore, the results of the present study can not be extrapolated to the total female population of our country. However, since the outpatient clinic is a referral one the results of the present study give us important information regarding the prevalence of HPV infection among young and middle aged women.

## Conclusions

In conclusion, the prevalence of HPV in women attending an outpatient clinic is high showing the importance of the early screening as well as the necessity of preventive measures. The number of sexual partners and alcohol consumption were the most significant risk factors for HPV infection, followed by young age and lower income. Since, the vaccine against the most prevalent and high risk HPV subtypes is in use in policies regarding prevention of HPV infection might help to reduce the risk of infection and cervical cancer. However, larger epidemiological studies in different regions of our country are needed in order to report the accurate prevalence of HPV infection.

## Competing interests

The authors declare that they have no competing interests.

## Authors' contributions

All authors participated in the collection, analysis, interpretation of data and writing of the paper. All authors read and approved the final manuscript.

## Pre-publication history

The pre-publication history for this paper can be accessed here:

http://www.biomedcentral.com/1471-2334/10/27/prepub

## Supplementary Material

Additional file 1 **Table S1**. The demographic characteristics of the study population [the variables are expressed as n (%)]. Click here for file

Additional file 2 **Table S2**. Univariate and multivariate logistic analysis: the association between various parameters with HPV infection in the study population. Click here for file
