# Supplementary material for: Prevalence of HPV infection among Greek women attending a gynecological outpatient clinic
Source: BMC Infect Dis. 2010 Feb 15;10:27. doi: 10.1186/1471-2334-10-27 (PMC2829020; doi:10.1186/1471-2334-10-27)
Supplement: Additional file 1 — Table S1. The demographic characteristics of the study population [the variables are expressed as n (%)]. [file 1471-2334-10-27-S1.DOC]

**Table S1.** The demographic characteristics of the study population [the variables are expressed as n (%)].

|  | HPV positive women | HPV negative women | P value |
| --- | --- | --- | --- |
| N | 51 | 174 | - |
| Age ± SD (yrs) | 28.5 ± 6.7 | 31.3 ± 6.7 | 0.01 |
| Monthly income (Euros)  ≤ 1000  1000-1999  2000-2999  ≥ 3000 | 21 (41.2)  13 (25.5)  7 (13.5)  10 (19.8) | 30 (17.2)  59 (33.0)  39 (22.4)  46 (26.4) | 0.004 |
| Educational level  Low  High | 14 (27.5)  37 (72.5) | 57 (32.8)  117 (67.2) | 0.51 |
| Nationality  Greek  Other | 45 (88.2)  6 (11.8) | 163 (93.7)  11 (6.3) | 0.29 |
| Marital status  Never married  Married  Divorced | 37 (72.5)  11 (21.6)  3 (5.9) | 81 (46.6)  91 (52.3)  2 (1.1) | 0.001 |
| Number of full term pregnancies  0  1  2-4 | 39 (76.5)  7 (13.7)  5 (9.8) | 109 (62.6)  26 (14.9)  39 (22.5) | 0.07 |
| Methods of contraception  None  Condom  Other | 15 (29.4)  31 (60.8)  5 (9.8) | 62 (35.6)  101 (58.0)  11 (6.4) | 0.67 |
| Age of first sexual intercourse (yrs)  ≤ 15  16-19  ≥ 20 | 5 (9.8)  30 (58.8)  16 (31.4) | 14 (8.1)  102 (58.6)  58 (33.3) | 0.87 |
| Total number of sexual partners  1  2  3-5  ≥ 6 | 2 (3.9)  11 (21.6)  22 (43.1)  16 (31.4) | 51 (29.3)  37 (21.6)  66 (37.9)  20 (11.2) | <0.001 |
| Number of sexual partners during last year  0  1  ≥ 2 | 4 (7.8)  33 (64.7)  14 (27.5) | 12 (6.8)  139 (79.8)  23 (13.4) | 0.11 |
| Number of sexual intercourses monthly  <2  2-4  5-8  ≥ 9 | 11 (21.6)  7 (13.7)  15 (29.4)  18 (35.3) | 28 (16.1)  36 (20.7)  51 (29.3)  59 (33.9) | 0.64 |
| History of sex transmitted disease  No  Yes | 23 (45.1)  28 (54.9) | 94 (54.0)  80 (46.0) | 0.26 |
| History of previous HPV infection  No  Yes | 39 (76.5)  12 (23.5) | 162 (93.2)  12 (6.8) | 0.99 |
| Last test PAP  Never  ≤ 1 year  > 1 year | 7 (13.7)  30 (58.8)  14 (27.5) | 11 (6.3)  119 (68.4)  44 (25.3) | 0.19 |
| Smoking status  Non or ex smoker  Current smoker | 23 (45.0)  28 (55.0) | 114 (65.5)  60 (34.5) | 0.01 |
| Alcohol consumption  No  Yes | 18 (35.2)  33 (62.8) | 97 (55.7)  77 (44.3) | 0.01 |
