# Supplementary material for: Prevalence of HPV infection among Greek women attending a gynecological outpatient clinic
Source: BMC Infect Dis. 2010 Feb 15;10:27. doi: 10.1186/1471-2334-10-27 (PMC2829020; doi:10.1186/1471-2334-10-27)
Supplement: Additional file 2 — Table S2. Univariate and multivariate logistic analysis: the association between various parameters with HPV infection in the study population. [file 1471-2334-10-27-S2.DOC]

**Table S2.** Univariate and multivariate logistic analysis: the association between various parameters with HPV infection in the study population.

|  | **Univariate analysis** | | | **Multivariate analysis** | | |
| --- | --- | --- | --- | --- | --- | --- |
|  | **Odds ratio** | **95% Confidence Intervals** | **P-value** | **Odds ratio** | **95% Confidence Intervals** | **P-value** |
| Age | 0.94 | 0.89-0.98 | 0.01 | 0.93 | 0.87-0.99 | 0.03 |
| Monthly income (high vs. low) | 0.65 | 0.48-0.89 | 0.007 | 0.63 | 0.44-0.89 | 0.01 |
| Marital status (married vs. single/divorced) | 0.37 | 0.18-0.73 | 0.004 | - | - | - |
| Number of full term pregnancies | 0.58 | 0.36-0.94 | 0.03 | - | - | - |
| Number of sexual partners | 2.17 | 1.50-3.15 | <0.001 | 2.16 | 1.44-3.25 | <0.001 |
| Smoking status (current vs. never/former) | 1.42 | 1.08-1.85 | 0.01 | - | - | - |
| Alcohol consumption | 2.27 | 1.18-4.35 | 0.01 | 2.19 | 1.04-4.63 | 0.04 |
